# Ayurveda, yoga, and acupuncture therapies in alleviating the symptom score among patients with spinal cord injury – A systematic review

**DOI:** 10.1016/j.jaim.2023.100749

**Published:** 2023-07-17

**Authors:** Sujatha KJ, Manjunath NK, Ahalya PG

**Affiliations:** a Division of Natural Therapeutics, SDM College of Naturopathy and Yogic Sciences, Ujire, D.K. Affiliated to RGUHS, Bangalore, Karnataka, India; b S-VYASA University, Bengaluru India; c SDM College of Naturopathy and Yogic Sciences, Ujire, D.K.Affiliated to RGUHS, Bangalore, Karnataka, India

**Keywords:** Acupuncture, Äyurveda, Panchakarma, Spinal cord injury, Yoga

## Abstract

**Background:**

Spinal cord injury (SCI) is the leading cause of motor and sensory abnormalities due to damage caused to any part of the spinal cord resulting from trauma, disease, or degeneration. Most of the disability caused will be irreversible with various systemic manifestations. Hence, management of SCI focuses on minimising disability, diminishing limitations due to impairment, and improving quality of life, emotional, and psychological aspects.

**Aim:**

This review is aimed at describing Ayurveda, Yoga, and Acupuncture therapies in the management of SCI as individual and integrated approaches for alleviating the symptom score in patients with SCI.

**Methods:**

The data was collected from six databases, including PubMed Central, the Cochrane Library, Google Scholar, Scopus, MEDLINE, and Grey Literature. The subjects in these studies were between the ages group 21–70 years and had been previously diagnosed with SCI and its clinical presentation. The interventions used in the selected studies incorporate Ayurveda (medicinal system of longevity) herbal medications, *Panchakarma* (five methods) treatment, diet, and yoga (mind-body medicine) therapy. Full-text publications in English, and research designs such as randomised controlled trials, case studies, review articles and cohort studies were included. Letter to the editor, study protocol, animal trials, and in vitro studies were excluded.

**Results:**

216 records were identified using keywords such as spinal cord injury, Äyurveda, Acupuncture, païca karma, rehabilitation, and yoga. After applying inclusion and exclusion criteria, 28 articles were selected for synthesis, which contain 12 case studies, 12 literature review articles, 2 randomised controlled trials, 1 cohort study, and 1 meta-analysis.

**Conclusion:**

The integration of Ayurveda management, including P*anchakarma*therapy and Ayurveda medications, with other alternative therapies like Acupuncture, Yoga, and Rehabilitation improved muscle strength, quality of life, range of motion, and neuronal function, and reduced depression, stress, and pain with symptom scores.

## List of abbreviations:

1. SCI: Spinal Cord Injury
2. CNS: Central Nervous System
3. RCT: Randomized Controlled Trial
4. PRISMA: Preferred Reporting Items for Systematic Reviews and Meta-Analyses
5. DMD: Duchenne Muscular Dystrophy

## 1 Background

Spinal cord injury (SCI) is an immediate and devasting condition, that causes damage to the spinal cord resulting in necrosis which leads to loss of conduction of impulses from the brain to the periphery, with an incidence of 40–80 cases per million population, where 90% are due to traumatic etiology. The prevalence of acute traumatic SCI is estimated to be 236 per million in India [1]. The physical disability arises from the location in descending tract, where the connection between synaptic input and output is disorganized resulting from trauma, degeneration, or any disease (cancer) [2,3]. The impact of SCI on sensory, motor, and autonomic functions relies on the location and extent of damage and unfortunately, the adult central nervous system (CNS) is unable to allow considerable axon development and regeneration of lengthy fiber tracts, as well as the adult CNS's limited capability to replace neurons, this functional impairment is mostly irreversible [4,5]. The pathophysiology of SCI is considered to be biphasic. The mechanical force injures the spinal cord, resulting in primary damage. Edema, ischemia, inflammation, cytokine production, free radical damage, glial scar formation, apoptosis, and necrosis contributes to secondary injury [6]. People who have had a SCI are 2–5 times more likely to die prematurely than those who do not. The risk of death rises with the severity of the injury and is heavily impacted by the availability of prompt, high-quality medical care immediately, without delay. Axon regeneration and functional recovery following SCI are influenced by a variety of inherent and extrinsic variables. Individually targeted therapeutic techniques will not be enough for substantial functional restoration [7,8]. As a result, spinal cord injury rehabilitation is a multi-dimensional, complicated condition that will need a combination of treatment techniques [9]. The key objectives of SCI rehabilitation are to minimize disability, alleviate the limitation of the impairment, to improve the quality of life, emotional and psychological aspects.

Ayurveda is a holistic approach, which regards physical, mental, and spiritual entities for diagnosis and management. The universe, according to Ayurveda, is made up of *pancha* *mahabhut*a (five elemental combinations) including, *Akash* (Ether), *Vayu* (Air), *Teja* (Fire), *Apa* (Water), and *Prithvi* (Earth). At all scales of life and in both organic and inorganic entities, the five elements may be seen in the material universe [10]. In Ayurveda medicine, there are srotas (channels) that transport fluids, and these channels may be opened up with oil massage and svedana (fomentation) [11]. The disease is considered to be caused by unhealthy or clogged channels. The tridosha (three bodily humors) that governs all bodily processes and maintains physiological and psychological balance includes, *Vata* (space or air, symbolizes the nervous system), *Pitta* (fire, symbolizes enzymes), *Kapha* (earth and water, symbolizes mucus) [12]. A balanced condition of the tridoña brings equilibrium and health; an imbalance between the physical and mental *dosha,* whether it's a *vriddhi* (excess) or a *kshaya* (deficiency), emerges as a symptom or indication of sickness [13]. Improvements in SCI have been shown with Ayurveda comprehensive management, which includes a few rehabilitation techniques as well as oral Ayurveda medicines [14]. Hence in this review, various Ayurveda therapies and their impact on disabilities due to SCI are described.

## 2 Methods

### 2.1 Study strategy and design

This study was conducted and reviewed according to Preferred Reporting Items for Systematic Reviews and Meta-Analyses (PRISMA) guidelines. The data was collected from 5 databases including PubMed Central, Cochrane Library, Google Scholar, Scopus, MEDLINE, and Grey literature using keywords Spinal cord injury, Ayurveda, *panchakarma*, and rehabilitation from 2003 to 2021. Full-text publications in English, and research design such as randomized controlled trials, case studies, review articles, and cohort studies were included. Letter to editor and study protocol were excluded. Studies with animal trials and studies carried out in vitro were not included. After applying inclusion and exclusion criteria 28 studies were selected for review.

### 2.2 Study population and intervention

**Fig. 1:**
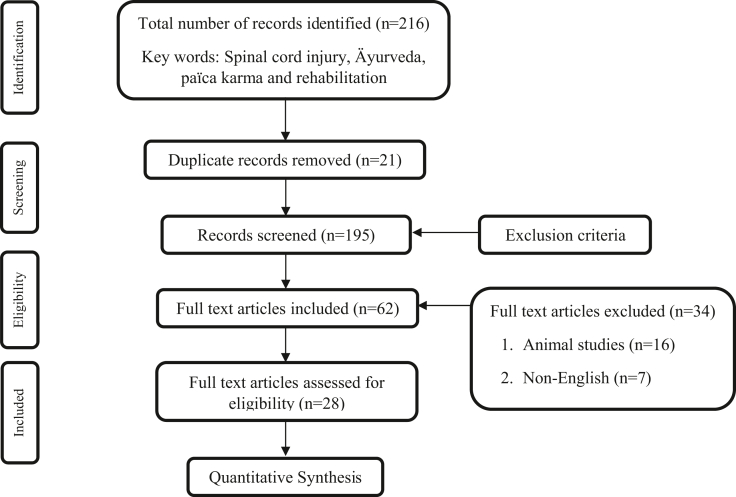
Flow diagram according to PRISMA guidelines.

The studies selected contained a population between 21 and 70 years of age, who were diagnosed previously with SCI and other systemic manifestations of SCI. All the study subjects were found to have normal higher mental functions. Studies with Ayurveda management for SCI and its manifestations were included. The interventions used in the selected studies incorporate, Ayurveda herbal medications, *Panchakarma* treatment, diet, and yoga therapy. This is shown in Fig. 1.

### 2.3 Outcome measures

General examination, a systemic examination was assessed for recording the symptom score, Visual analogue scale, Oswestry Low Back Pain Scale, and Neck disability score were assessed for pain and degree of disability, X-ray, and MRI were assessed for noting the structural changes before and after the intervention.

## 3 Results

In this review, 216 records were identified using the keywords Spinal cord injury, Ayurveda, *panchakarma* and rehabilitation. After applying inclusion and exclusion criteria and excluding the duplicate records (n = 21), 62 articles were selected of which 16 animal studies, 7 non-English studies, and 11 in-vitro studies were excluded and final synthesis was done for 28 eligible articles (Fig. 1). The articles selected for review include Ayurveda management (Ayurveda herbal medications, Panca karma treatment, Diet), Acupuncture and Yoga therapy for SCI and its systemic manifestations which contains 12 case studies, 12 literature review articles, 2 randomised controlled trials, 1 cohort study, and 1 meta-analysis. In Table 1 the article details along with the source and keywords and the design of the study are explained and in Table 2 intervention used, parameters assessed, and the conclusion is explained. The whole SCI and intervention for SCI manifestations are also reviewed in detail.

**Table 1:** Overview of characteristics of the records.

S/no	Title	Author and year	Keywords	Journal	Assessment	Duration of the study	Design of the study
1	Ayurvedic treatment of chronic fatigue syndrome—a case report	Sivarama prasad vinjamury et al., 2005	Not Applicable	Alternative therapies	• Modified fatigue impact scale	2 months	Case study
2	Clinical study to evaluate the efficacy of yapana basti and vanari gutika in the management of klaibya w.s.r. to erectile dysfunction	Mukesh kumar et al., 2017	Kalaiya sthiradipanchamooladi yapana basti, vanari gutika	International journal of ayurvedaand pharma research	• International Index of Erectile Function 15 items Objective parameters (semen analysis)	Not Applicable	Open label, randomized, comparative, interventional study using pre-test and post-test design
3	Evaluation of combined efficacy of nirgundi patra pinda swedana, greeva basti and matra basti in the management of cervical spondylosis: a case report	Achala r kumawat et al., 2019	Fomentation, shankara sweda, navarakhizi, shashtikashali,vatavyadhi.	World journal of pharmaceutical research	• Neck Disability Index.• Visual Analogue Scale • Range of Movement at ervical Spine.	14 days	Case study
4	Ayurvedic management in cervical spondylotic myelopathy	Sarvesh kumar singh; 2016	Ayurvedic treatment,cervical spondylotic myelopathy, greevastambha	Journal of ayurveda and integrative medicine	• Chile's modified Japanese Orthopaedic Association (mJOA) score • Range of motion	30 days	Case report
5	A review on concept of grivasandhigatavata and its management wsr cervical spondylosis	Pinkee gautam et al., 2019	Griva-sandhigatavata, cervical spondylosis, management, nidana	Journal of drug delivery and therapeutics		Not Applicable	Review article
6	Clinical evaluation of nasya karma in cervical spondylosis: case series	Sangeeta r. Tanwar et al., 2021	Cervical spondylosis, lifestyle disorders, nasya, panchakarma, urdhvajatrugata vikara, vata vyadhi	Indian journal of health sciences and biomedical research	• VAS (Visual analogue scale) • NDI • range of movements	7 days	Case series
7	Management of a case of lumbar stenosis with ayurvedic intervention	Kshirod kumar ratha; 2016	Low back pain, lumbar stenosis, panchakarma, katigraha.	Asian journal of pharmaceutical and clincal reasearch	• Grading of chronic (LBP) pain • Femoral nerve stretch • Straight leg raise	2 months	Case study
8	Neuroprotective effect of ashwagandha (roots of withania somnifera): the rejuvenator	Vandita singh; 2017	Alzheimer's disease, neurodegenerative diseases, parkinson's disease, withania somnifera	The canadian journal of clinical nutrition		Not Applicable	Review article
9	Review on nutritive ayurveda bolus fomentation: shasthikashali pinda swedana	Nirmal bhusal; 2017	Fomentation, shankara sweda, navarakhizi, shashtikashali,vatavyadhi.	Acta velit	• VAS • Left knee joint circumference • Left Knee joint ROM	Not Applicable	Review article
10	A comparative efficacy study of the panchtikta ghrita matra vasti and panchtikta ghrita marsha nasya in cervical spondylosis	Punam sawarkar et al., 2021	Panchatikta ghrit, matra basti, nasya, cervical spondylosis.	International journal of legal medicine	• Neck disability index • CBC • ESR • X RAY	28 days	Open randomized parallel comparative clinical study
11	Spinal injury – induced paraplegia improvement after panchakarma	Rajkala p. Patil et al., 2018	Adharangaghata, panchakarma, yapana basti	Journal of research in ayurvedic sciences	• Spinal cord independent score	3 months	Case report
12	Effect of mustadi rajayapana vasti and selected treatment regimen in the management of spinal cord injury- a case study	Manori jansz et al., 2021	Mustadi rajayapana vasti, panchakarma, spinal cord injury	International journal of ayush case reports	• Spinal Cord Independence Measure Score • Medical Research Council (MRC)grading scale in muscle power	82 days	Case study
13	Ayurvedic approach in the management of spinal cord injury: a case study	Sarvesh kumar singh et al., 2015	Matra basti, Mustādi yāpana basti, patient centered outcome, quadriplegia, spinal cord injury, stem cells therapy	Ancient science of life	• Spinal Cord Independence Measure (SCIM-III) scoring	2 months20	Case study
14	Contribution of ayurveda in management of spinal cord injury (sci) induced paraplegia	Punam sawarkar; 2017	Not Applicable	Best practices in panchakarma	–	20 days followed by 17 days with a gap of 8 days	Case study
15	Rehabilitative potential of ayurveda for neurological deficits caused by traumatic spinal cord injury	Sanjeev rastogi; 2014	Practice-based evidence, patient centered outcome, quadriplegia, rehabilitation, spinal cord injury	Journal of ayurveda & integrative medicine	–	3 months	Case study
16	Acupuncture for symptomatic gastroparesis	Kun hyung kim; 2018	Not Applicable	Cochrane Database Syst Rev	• Gastroparesis Cardinal Symptom Index • Quality of life	Not Applicable	Review article
17	Ayurveda in treatment of bone disorders in human: a review	Subha ganguly; 2015	Ayurveda, bone disorders	Int j ayu pharm chem	–	Not Applicable	Review article
18	Current concepts in neural regeneration- a systemic review	K. Gayathri devi et al., 2017	Neural regeneration, nervous system	Research j. Pharm. And tech	–	Not Applicable	Review article
19	Therapeutic effects of raja yoga - a review	K shipra rajoria et al., 2017	Biomedical, meditation, raj yoga, review	Indian journal of traditional knowledge	–	Not Applicable	Review article
20	Role of rajayapana basti with reference to duchenne muscular dystrophy: a review	Abeynayake pemadasa; 2016	Duchenne muscular dystrophy, adibala pravrit mamsa-vata-kshaya, panchkarma, rajayapana basti	Int. J. Res. Ayurveda pharm.	• range of joint motion • Muscle strength	Not Applicable	Review article
21	First episode of acute CNS inflammatory demyelination in childhood: prognostic factors for multiple sclerosis and disability	Yann mikaeloff et al., 2003	Not Applicable	The journal of pediatrics	• MRI	2.9 ± 3 years	Cohort study
22	Management of hemangioblastoma of brain with ayurveda and yoga: a case report	Umesh kumar sapra et al., 2021	Aavarana, cystic lesion, hemangioblastoma, vata vyadhi, yoga	Journal of ayurveda case reports	• MRI • CT	10 months	Case report
23	A review on co-relation between swedan karma and thermotherapy in pain management	Akshaya g. Patil et al., 2018	Swedan karma and thermotherapy in pain management.	Journal of vishwa ayurved parishad	Not Applicable	Not Applicable	Review article
24	Physical fitness training for stroke patients	David h saunders et al., 2020	Not Applicable	The cochrane collaboration.	• Pooled functional scales • VO2 peak • 6-Minute Walk Test • Berg Balance Scale	Not Applicable	Review article
25	A review article on common pathological conditions affecting trimarma in light of classical insights	Tripathi richa et al., 2018	Trimarma, hriday, shira, basti, pathological conditions	International journal of ayurveda and pharma research	Not Applicable	Not Applicable	Review article
26	Respiratory muscle training for cervical spinal cord injury	David j berlowitz et al., 2014	Not Applicable	The cochrane collaboration.	• vital capacity • maximal inspiratory pressure • maximal expiratory pressure	Not Applicable	Review article
27	Majjabasti in low back pain	Meena shamrao deogade; 2017	Not Applicable	Best practices in panchakarma	• VAS	2 weeks	Case study
28	The natural history of complete spinal cord injury: a pooled analysis of 1162 patients and a meta-analysis of modern data	Najib e. El tecle et al., 2017	American spinal injury association; asia; complete spinal cord injury; conversion; meta-analysis	J neurosurg spine	• American Spinal Injury Association (ASIA) grading system	Not Applicable	Meta-analysis

**Table 2:** Overview of findings of the Records.

S/no	Parameters	Intervention	Findings
1	MFIS Fatigue score	Ayurveda drugs, Diet and Breathing exercises	The total MFIS score fell from 63 to 27. The physical subscale dropped from 27 at the start to 12 after two months. The cognitive subscale decreased from 30 at baseline to 12 at the end of the study. The psychosocial scale went from 6 to 2 on the scale.
2	International Index of Erectile Function 15 items Objective parameters (semen analysis)	Group A *- Yapana Basti* Group B - *VanariGutika* Group C - Both	The total effect of subjective criteria in groups A, B, and C was very significant, with 55.55 percent, 34.78 percent, and 94.44 percent, respectively. In the semen analysis, group-c had a highly significant result with 42.34 percent (semen volume), 15.97 percent (Sperm count), and 45.01 percent (immotile sperm) correspondingly.
3	VAS. Range of Movement at Cervical Spine. Neck disability score	*Nirgundi Patrapinda svedana* followed by *Greeva Basti and Matra Basti* with *Dashmoola Taila*	Cervical spondylosis is a *KaphaVata* dominating disease, and *Nirgundi patr*a pinda svedana pacifies *Vata* linked with *kapha*, followed by *Greeva Basti*, which improves local blood circulation and strengthens local tissues and nerve roots, alleviating pain and related symptoms. Furthermore, *Matrabasti* promotes sleep while also lowering mental anguish induced by pain and disturbed sleep.
4	Pain, giddiness, neck stiffness, neck motion, power and reflexes of upper and lower limbs, MRI	*Shalishastika Pinda Svedana, Mustadi Yapana Basti,* Ayurveda drugs	The MRI showed a significant improvement in ligamentum flavum hypertrophy, which caused spinal canal constriction and spinal cord compression at many levels, most notably at C-3-4, with thinning of the spinal cord at this level and cord edema, as compared to prior MRIs. The discomfort had vanished. There was no giddiness in the patient. Neck stiffness had significantly decreased. Range of motion of neck was normalised.
5	Not Applicable	*Panchakarma, Pathya Ahara* and certain yogic procedures	For the therapy of *Sandhigatavata*, Acharya Sushruta and Acharya Vagbhatta have defined *Snehana, Upanaha, Agnikarma, Bandhana, Mardana,* and *Svedana* as a distinct line of treatment.
6	Clinical symptoms of cervical spondylosis;Visual Analogue Scale(VAS); Neck disability index (NDI); Range of movement.	Local A*bhyanga; Mridu Sweda; Uttanasya Shyanasya* with *Pralambita Shirsah Kinchit*	Improvement with numbness (75 percent), discomfort (73.68 percent), stiffness (66.67 percent), giddiness (60 percent), tingling feeling (50 percent), and headache (18 percent) was noted after therapy was completed. The VAS and NDI both decreased by 75.45% and 76.92%, respectively. The range of motion was also much improved, with full relief in neck flexion and lateral rotation, as well as 90% improvement in right lateral flexion, 88.89 percent improvement in left lateral flexion, and 60% improvement in extension.
7	Grading of chronic low back pain and local examination of spine	*Sarvang Abhyaìgaand Patarpindasweda: Kativasti with Ksheerabala Taila; Virechana-Gandharvahastadi Eranda Taila;* Ayurveda drugs	Pharmacological activities include anti-inflammatory, analgesic, antioxidant, and immune-stimulant properties. All of the treatments performed in this case were targeted at decreasing pain, stiffness, improving muscular power, strengthening spinal muscles, and correcting the curvature of the spine.
8	Not Applicable	*Withania somnifera* roots	Roots of *W. somnifera* exhibit great potential as a safe and effective neuroprotectant. It might be a good neuroprotective treatment for Alzheimer's, Parkinson's, anxiety, stress, cognitive, and other nervous system problems. So far, no harmful or adverse effects have been recorded for this medication.
9	Not Applicable	*Shasthikashali Pinda Sweda*	*Pinda Shashtikashali Sweda* is an Ayurveda therapy that aids in improving tissue strength, immunity, and nutrition, preventing degeneration and inflammation, and treating pain and swelling associated with Arthritis, Neuromuscular and Musculoskeletal diseases
10	Neck Disability Index; Subjective Parameters; X-ray cervical spines	Group A- *Panchtikta Ghrita Matra Vasti;* group B- *Panchtikta Ghrita Marsha Nasya.*	Due to the short timeframe of the intervention and research, there was no major improvement in radiological results in the cervical X-ray spines. Both groups showed a significantly improvement in decreasing cervical spondylosis symptoms and minimizing the neck disability score. It was also statistically proven that *Nasya* with *Panchtikta Ghrita* is more effective than *Matra Vast*i with *Panchtikta Ghrita* when comparing the two groups.
11	Assessment of muscle strength; Assessment of reflexes; ASIA Score	*Sarvang Abhyanga-Swedana; Rajyapana Basti;* Physiotherapy; Äyurveda medications	In L1-L2 SCI, *pancha karma* treatments are helpful in achieving functional grasp. From ASIA grade A to ASIA grade D, the patient's condition has improved. Motor scores increased from 51 to 88 on a scale of 100.
12	Spinal Cord Independence Measure Score; Reflex grading scale; grading scale for muscle power and writing ability by Medical Research Council	*Vasti Karma;* Ayurveda medications	Before therapy, the net SCIM score was 15, and after treatment, it was 65. Micturition and defecation were restored by the patient. In both extremities, muscle power increased from grade 1 to grade 4.
13	Clinical examination, Spinal Cord Independence Measure (SCIM-III)	*Panchakarma,* Ayurveda oral medications	Through use of *Panchakarma* treatments and Ayurveda management to treat stable SCI created an ideal solution to manipulate neurological impairments. This effect might be caused by many processes, including acetoacetate's suppression of the vesicular glutamate transporter, higher adenosine levels, and increased activity of the ATPsensitive K+ channels, which decrease excitement.
14	Hematological investigation, MRI, X ray of lumbo sacral spine, Clinical examination	*Panchakarma,* Ayurveda medications	Ayurveda management can successfully address critical diseases like SCL Induced Paraplegia by enhancing the patients' quality of life. Monocarboxylic acid transporters, which are active during *Abhyaìgaand Vasti*, aid in the transfer of lactate, pyruvate, and ketone bodies across biological membranes and aid in neuroprotection, resulting in better recovery of neuronal function after SCI
15	Modified Barthel Index, Clinical Assessment	*Pinda Sweda, Greeva Vasti* *Shiro Vasti, Kaala Vasti,* Ayurveda oral medications	This therapy did not achieve total function independence in the instance, but it did significantly reduce dependency, as seen by the improved MBI score.
16	Not Applicable	Acupuncture	Acupuncture was used as part of an extensive treatment plan in the majority of the trials. Manual acupuncture stimulation and electrical stimulation produced equal results in terms of the proportion of individuals whose symptoms improved, according to subgroup analyses.
17	Not Applicable	*Abhya*n*gaand* Oral medications	*Guggul* and *kutki* are two herbs that are used to purify the bones and are particularly effective in curing kapha doña in the asthi dhatu. *Vata* pacification can be achieved by *abhyanga*, or self-massage with sesame oil, and *Dashamularishta.*
18	Not Applicable	Ayurveda oral medications	*Rasayana* herbs are well-known for their tissue-protecting properties. Curcumin has been shown to increase hippocampus neurogenesis in both children and adults, as well as a biological process that may improve brain plasticity and repair. The contractility of cardiomyocytes generated from embryonic stem cells was improved after treatment with herbal extract. The action of *Dhanvantar Kashaya* (a decoction of herbs with regeneration properties) on Wharton jelly mesenchymal stem cells has been observed (WJMSCs). The decoction enhanced the rate of proliferation, reduced turnover time, and postponed senescence.
19	Not Applicable	*Asana, Dhyana, Pranayama*	Yoga lowers salivary cortisol, blood glucose, blood pressure, plasma rennin, and urine nor-epinephrine and epinephrine levels throughout a 24-h period. Atherosclerosis is reversed, myocardial ischemia is reduced, and left ventricular hypertrophy is reduced as a result of these causes.In patients with chronic obstructive lung disease, yoga treatment reduces dyspnea-related discomfort and improves functional performance. Yoga can help with depression by increasing serotonin levels while decreasing monamine oxidase, an enzyme that breaks down neurotransmitters and cortisol.
20	Not Applicable	*Rajayapana Basti*	Due to its *Sadhyo Balajanana* and *Rasayana* effects, *Panchkarma* with *Rajayapana Basti* may be proven to have the most rational and cost-effective affect. It improves the quality of life and extends the lifespan of DMD patients. It instantly boosts physical strength and increases body power.
21	Kurtzke Disability Status Scale, MRI, Poser criteria, clinical examination	Not Applicable	The researchers looked at the predictive variables for a second attack and impairment in children who had a first episode of CNS demyelination. Patients with mental status changes had a reduced risk of a second bout of CNS demyelination than those who are older. Polysymptomatic and recurrent individuals have a greater risk of impairment.
22	General examination, systemic examination, MRI	Ayurveda oral medications, yoga	Hemangioblastoma in the brain has been linked to the Ayurveda concept of *Prana aavrita samana vata*. Ayurveda medicines, in combination with Yoga treatment, can help control the condition. It benefits patients by boosting their strength, removing toxins, cleansing circulation channels, and making the body and mind more steady and focussed.
23	Not Applicable (Patil VV. Recent Advances in Instrumentations and techniques of Pancha karma Therapy. DOI: 10.13140/RG.2.1.3471.0165)	*Swedan karma,* Thermotherapy	Since the blood in the *Swedan* region takes the heat away, any type of heat should be administered gradually to allow for vasodilation. The effects of thermotherapy on tissue metabolism, blood flow, inflammation, edema, and connective tissue extensibility help in pain management. *Swedan karma* corrects the function of the medadhatwagni and bhutagni, as well as the paka karma, resulting in srotomukhasodhana and abundant sweda generation. Exudate displacement relieves pain and calms muscle spasms.
24	Death, Disability, Mixed training interventions, Health status and quality of life:, Mood, Physical function and fitness, Mobility and Adverse effects	Cardiorespiratory training interventions, Resistance training interventions, Mixed training interventions	Cardiorespiratory training and, to a lesser extent, mixed exercise, decrease impairment during or after standard stroke treatment, possibly through better mobility and balance. It has been hypothesised that increasing VO2 peak following cardiorespiratory exercise reduces the incidence of stroke hospitalisation by 7%. Despite being a crucial outcome of interest for patients, cognitive function is understudied.
25	Not Applicable	Not Applicable	The *Prana* means vital breath resides in *Basti, Hriday and Shira*. As a result, every effort should be made to safeguard them*. Trimarma'*s external injuries may be the result of unavoidable events, but internal injuries are the most prevalent cause.
26	Respiratory complications, dyspnoea, and VC, Measures of respiratory muscle strength (maximal inspiratory pressure (MIP), maximal expiratory pressure (MEP), forced expiratory volume in 1 s (FEV1), and quality of life.	Respiratory muscle training (RMT)	RMT may increase VC and maximal respiratory pressures (MIP and MEP) for people with cervical SCI. However, the effect size for all three outcomes was small and there was no evidence of carryover beyond the training period. Some evidence shows that inspiratory muscle training could improve respiratory function and decrease dyspnoea.
27	Oswestry Low Back Pain Scale.	*Dashamoola Nirgundi kwath Nadisweda* followed by *Majja basti*	The *shukra, rasa, shleshma, meda,* and *majja* are all enhanced by *majja.* It helps with neurological diseases, muscle and tissue numbness, back and lumber stiffness, and all vitiated vata-related ailments. Snehana svedana improves blood circulation and reduces spasms and stiffness in the local area. *Majjabasti* nourishes the saptadhatu and strengthens the muscles, therefore relieving pain and providing symptomatic relief to the patient.
28	American Spinal Injury Association grade A - ASIA A	Not Applicable	From pooled data from prospective trials and observational series, the total rate of conversion of ASIA grade A SCIs is 28.1 percent and looks to be higher.

## 4 Discussion

This systematic review was undertaken to provide the evidence base for recommendations of alternative therapies in the management of pain in SCI patients. The up-regulation of specific inflammatory molecules following damage, which leads to gliosis, is one of the longer reaction times to get recovered completely from SCI. Modern medicine uses a variety of surgical techniques, stem cell implantation therapy, and other medicinal therapies, however, they are all limited [15]. Treatment of secondary damage with Ayurveda intervention may aid in healing from SCI and its presentation [16]. Ayurveda medications like rasäyana herbs are well-known for their tissue-protecting properties. Curcumin has been shown to increase hippocampus neurogenesis in both children and adults, as well as a biological process that may improve brain plasticity and repair. The contractility of cardiomyocytes generated from embryonic stem cells was improved after treatment with the herbal extract. The action of dhanvantari kashaya (a decoction of herbs with regeneration properties) on Wharton jelly mesenchymal stem cells has been observed (WJMSCs). The decoction enhanced the rate of proliferation, reduced turnover time, and postponed senescence [17,18]. The pharmacological effects of these medications include anti-inflammatory, analgesic, antioxidant, and immune-stimulant properties. All of the treatments performed in this case were targeted at decreasing pain, and stiffness, improving muscular power, strengthening spinal muscles, and correcting the curvature of the spine [19].

*Panchakarma* has many therapeutic advantages, including cleansing, better circulation, and pain reduction. *Panchakarma*'s effectiveness has also been demonstrated in the treatment of pain-related illnesses. It relieves tension, relaxes the body, lowers pain, and provides a soothing effect while improving circulation [20,21]. The *shukra* (reproductive fluid or semen) rasa (plasma or lymph fluid) çleçma (mucus), meda (fat), and majja (bone marrow) are all enhanced by *majja basti* (therapeutic enema) [22]. It helps with neurological diseases, muscle and tissue numbness, back and lumber stiffness, and all vitiated Vata -related ailments. *Snehana svedana* (heat therapy after oil or lubricant application) improves blood circulation and reduces spasms and stiffness in the local area. *Majja basti* nourishes the *saptadh*a*tu* and strengthens the muscles, therefore relieving pain and providing symptomatic relief to the patient.

*Vasti* is the best treatment for vitiated Vatadosha. *Yapana vasti* can support life and promote longevity. Abhyaìga and svedana are considered as external therapeutic procedures that mitigate vitiated Vatadosha. The immense number of nerves which are located in the enteric nervous system can be nourished easily and quickly due to the *Sadyaobalajanana* and *Rasayana* effect of *Vasti* and *Abhyanga* [23].

Yoga is gaining popularity in modern medicine for its ability to improve health and well-being, and it has been studied in a range of clinical groups for both acute and chronic illnesses. Yoga is a safe and supportive mind–body practice that may simultaneously attenuate some of the negative psychological impacts of SCI and is beneficial as a stand-alone treatment or as adjuvant therapy in the treatment of a range of chronic illnesses in randomised controlled trials [24,25]. When people with post-stroke hemiparesis participated in a 10-week yoga study, their depression levels improved clinically when compared to others who did not participate. Thus, Yoga Practice results in increased mindfulness from preintervention to postintervention, with an increased capacity to observe and not react to immediate physical and emotional experiences [26]. Yoga benefit patients by boosting their strength, removing toxins, cleansing circulation channels, and making the body and mind steadier and more focused.

Acupuncture and rehabilitation training was shown to be more beneficial than rehabilitation training alone at reducing postvoid residual (PVR) urine volume for chronic urinary retention (CUR) due to SCI.

Ayurveda management can successfully address critical diseases like SCI-induced paraplegia by enhancing the patient's quality of life. Monocarboxylic acid transporters, which are active during *Abhyanga* and *Vasti*, aid in the transfer of lactate, pyruvate, and ketone bodies across biological membranes and aid in neuroprotection, resulting in better recovery of neuronal function after SCI [27]. Hence, from this review, it is evident that Ayurveda management integrated with conventional intervention is proven to be beneficial for patients with SCI and its manifestations.

## 5 Conclusion

In this study, 28 articles were reviewed, in which, the integration of Ayurveda management including *panch* *karma* therapy and Ayurveda medications along with conventional management showed improvement in muscle strength, Quality of life, range of motion and neuronal function and reduced depression, stress, and pain. The Ayurveda treatments when administered with yoga and acupuncture, help in better recovery in symptom score, and pain through improving local blood circulation, strengthening local tissues, and preserving nerve degeneration.

## Funding

The research has been self-funded.

## Author contribution

**Dr. Sujatha Dinesh:** Conceptualization, Visualization, Methodology, Funding Acquisition, Writing - Review & Editing; **Dr. Manjunath:** Data curation, Formal analysis, Validation; **Dr. Ahalya:** Writing- Original draft preparation.

## Declaration of competing interest

There is no potential conflict of interest during the review.
